# Combined exercise and nutrition intervention for spinal sarcopenia: A pilot study protocol

**DOI:** 10.1097/MD.0000000000026421

**Published:** 2021-06-18

**Authors:** Sang Yoon Lee, Jinhee Park, Dong Hyun Kim, Jae-Young Lim

**Affiliations:** a Department of Rehabilitation Medicine; b Department of Radiology, Seoul National University College of Medicine, SMG-SNU Boramae Medical Center, Seoul; c Department of Rehabilitation Medicine, Seoul National University College of Medicine, Seoul National University Bundang Hospital, Seongnam-si, Gyeonggi-do, Republic of Korea.

**Keywords:** nutritional support, paraspinal muscles, resistance training, sarcopenia, spine

## Abstract

**Introduction:**

Spinal sarcopenia is a multifactorial disorder associated with the atrophy of and fatty changes to the paraspinal muscles. We previously developed the concept of spinal sarcopenia in community-dwelling older adults and investigated the association between conventional sarcopenic indices and spinal sarcopenia. However, interventional studies of spinal sarcopenia are lacking. This pilot study will aim to evaluate the effectiveness of a combined exercise and nutrition intervention for treating spinal sarcopenia.

**Methods and analysis:**

This open-label single-arm prospective study will include 35 community-dwelling older women who were diagnosed with spinal sarcopenia in our previous cohort study. The 12-week combined intervention will consist of back extensor strengthening exercise and nutritional supplementation. The primary outcome of this study will be isometric back extensor strength after the 12-week intervention. All functional and radiographic outcomes will be measured at 0, 12, and 24 weeks post-intervention. The data will be analyzed using the intention-to-treat principle.

## 1 Introduction

### 1.1 Background

Spinal sarcopenia is a multifactorial disorder associated with the atrophy of and fatty changes to the paraspinal muscles.^[1]^ With age, the cross-sectional area of the paraspinal muscles tends to decrease, whereas the fat infiltration increases.^[2]^ Paraspinal muscle atrophy significantly contributes to spinal disorders in terms of back pain, low quality of life (QoL), and spinal dysfunction.^[3]^ It also leads to the loss of lumbar segmental stability, resulting in recurrent low back pain.^[4]^ Spinal sarcopenia is correlated with spinal sagittal imbalance due to insufficient compensation by flattening thoracic kyphosis (TK) in patients with a spinopelvic mismatch.^[5]^

The development of new medical technologies and pharmacological treatments for sarcopenia, ranging from prevention to treatment and rehabilitation, is an immediate task in ageing societies. In particular, although the development of new drugs is urgently required for the medical access and treatment of sarcopenia, no new drug has yet passed phase III or higher clinical trial testing.^[6]^ Therefore, resistance exercise and nutritional supplementation,^[7]^ the current first-line interventions for the prevention and treatment of sarcopenia, should be applied equally in cases of spinal sarcopenia.

In a preliminary study in 2019, we recruited elderly community-dwelling individuals (SarcoSpine cohort).^[1]^ In that study, lumbar spine magnetic resonance imaging (MRI) was performed for the quantitative evaluation of the lumbar extensor muscles (LEM) of all subjects, while several functional tests of the spine, including isometric/isokinetic back muscle strength and back performance tests, were conducted. The second wave of the cohort is expected to begin in the second half of 2021. Here, we describe a planned pilot study of a combined exercise and nutrition intervention for treating spinal sarcopenia in elderly women whose spinal muscle mass is in the lower 50% of this cohort. This study will aim to determine whether completing 12 weeks of the combined intervention has a therapeutic effect on spinal sarcopenia in elderly women.

### 1.2 Objectives

1. To develop the combined exercise and nutrition intervention for spinal sarcopenia in elderly women.
2. To determine the feasibility and effectiveness of treating spinal sarcopenia with 12 weeks of the combined intervention.

## 2 Methods

### 2.1 Trial design

This will be an open-label single-arm study of 35 community-dwelling older women in a single center (SMG-SNU Boramae Medical Center) with a 24-week follow-up period. The trial has been registered prospectively with the Clinical Trials.gov Registry (NCT04810312) prior to participant recruitment. Important protocol modifications will be communicated to the trial registry.

### 2.2 Participants and eligibility criteria

Community-dwelling older women (age: ≥65 years) who were diagnosed with spinal sarcopenia in our previous cohort study^[1]^ will be included. In the cohort study, a quantitative evaluation of the spinal extensor muscles was performed using dual-energy X-ray absorptiometry (DEXA) in the lateral direction, and the cutoff value of the lower 50% of measurements was 562 g. Therefore, we decided to deliver a combined exercise and nutrition intervention to 35 subjects whose spinal extensor muscle mass was <562 g in this planned pilot study. Sarcopenia and spinal function will be assessed before and immediately after as well as 12 weeks after completion of the intervention (for a total of 24-week study duration).

The exclusion criteria for this pilot study will be the same as those of the previous cohort study. If any of the following conditions are satisfied after participation in the previous study, a participant will be excluded: 1) moderate-severity low back pain (numeric rating scale score ≥5); 2) history of any type of lumbar spine surgery; 3) history of hip fracture surgery or arthroplasty of the hip or knee; 4) contraindications for MRI (such as a cardiac pacemaker, implanted metallic objects, and claustrophobia); 5) disorders of the central nervous system (such as stroke, parkinsonism, and spinal cord injury); 6) cognitive dysfunction (Mini Mental State Examination score <24); 7) communication disorder (such as severe hearing loss); 8) musculoskeletal condition affecting physical function (such as limb amputation); 9) long-term use of corticosteroids due to inflammatory disease; 10) malignancy requiring treatment within the previous 5 years; and 11) other medical conditions requiring active treatment. Subjects who refuse to participate in this study will also be excluded.

### 2.3 Combined exercise and nutritional intervention

All subjects of this study consist of a group with 2 to 3 people who will visit our institute every 2 weeks for a total of 12 weeks. In each session, participants will complete a 50-minute spinal extensor strengthening exercise program.^[8,9]^ Exercise intensity will be calculated as the number of seconds each exercise is maintained per set. The major muscle groups will be warmed up and cooled down for 5 minutes before and after each exercise session. The back extensor strengthening exercises will include McKenzie back extension (5 minutes); curl-up, side-bridge, and bird-dog (5 minutes each); plank (basic > elbow > 1-side) (10 minutes); and squatting (10 minutes). The back extensor strengthening exercises will be detailed in an exercise booklet so each participant can exercise at home 3 times a week (including 1 visit to the institution every 2 weeks), and we will increase the exercise intensity by 20% every 2 weeks. To improve exercise compliance, we will contact the study subjects via video call every week and check their participation.

For a nutritional supplement, each individual's typical calorie and protein intake will be calculated through the 24-hour recall method. With a goal of 1.2 g/kg daily protein intake,^[10]^ 1 liquid protein supplement and 1 energy bar (Selex core protein: Maeil Health Nutrition, South Korea) will be supplied per day for 12 weeks. Liquid protein and energy bar will contain 20 g of protein with 1830 mg of leucine per day.

### 2.4 Outcomes measures

The following outcomes will be measured before and immediately after the 12-week intervention, as well as 12 weeks after its completion for an overall 24-week study period (Table 1).

**Table 1:** Overview of the outcome measures and time points of assessment.

	Visit 0	Visit 1	Visit 2	Visit 3	Visit 4	Visit 5	Visit 6	Visit 7	Visit 8
	Screening	Week 0 (start point)	Week 2	Week 4	Week 6	Week 8	Week 10	Week 12 (endpoint)	Week 24
Eligibility	O								
Informed consent	O								
Demographic information	O								
Medical history	O								
Whole-body DEXA and BIA	O							O	O
Isometric and isokinetic back muscle strength	O							O	O
Whole spine X-ray (lateral)	O							O	O
Physical performance test	O							O	O
Nutritional assessment	O							O	O
L-S spine MRI								O	
Exercise education and nutritional supplement		O	O	O	O	O	O	O	

Isometric back muscle strength will be the primary outcome measure, which will be assessed using a handheld dynamometer (PowerTrack II; JTECH Medical, Salt Lake City, UT, USA). This will involve the participant standing in full extension midway between 2 vertically oriented anchor rails with their back to a wall, feet flat on the floor, and heels touching the wall. A belt will be looped through the anchor rails and secured firmly around the participant 1 cm below the anterior superior iliac spine to restrain movement and ensure participant contact with the wall during the test. To standardize the posture, the arms will be crossed over the chest with the fingertips level with the contralateral shoulders. The participant will be instructed to flex forward approximately 15° at the hips so the handheld dynamometer can be positioned posterior to the spinous process of the seventh thoracic vertebra. In this way, counterpressure will be provided by the fixed wall behind the back to prevent the introduction of variations in resistance by the examiner.^[11]^

The secondary outcome measures^[1]^ will be performed as follows:

1. *Isokinetic back muscle strength*
  The investigators will use an isokinetic dynamometer (Biodex Multi-Joint System; Biodex Corporation, Shirley, NY, USA) to measure the back extensor torque. Briefly, the examination will be performed by seating the patient comfortably in the device, fixing both the thighs and the back to the chair using a strap, and asking the patient to hold the handle placed near the chest for the measurement of the upper limb and hip joint motions. The dynamometer axis will be located on the anterior superior iliac spine of the patient's pelvis. All patients will be instructed to flex and extend the back 5 times at an angular velocity of 60°/sec as a warm-up before the examination. During the examination, the patients will be instructed to execute maximal-effort back flexion and extension 10 times at an angular velocity of 60°/sec. The back range of movement will be limited at 50° with 30° (−30°) of trunk flexion and 20° (+20°) of trunk extension relative to the anatomical reference position (0°).^[12]^ The device will measure the peak torque (Nm) and the peak torque per body weight (Nm/kg).^[13]^
2. *LEM mass*
  DEXA (Lunar iDXA for Bone Health; GE Healthcare, Schenectady, NY, USA) will be used to analyze LEM mass. Lateral whole-body scans will be taken according to the enCORE-based X-ray Bone Densitometer User Manual. The lateral positioner and the instructions below aim to position the lumbar spine straight and parallel to the scanner table: 1) place a pillow under the subject's head, 2) allow the subject to lie on the left side with the lower limb joint as comfortable as possible, 3) position the subject's back and hips flat against the positioner, and 4) position the subject's arms at 90° from the chest. After the lateral DEXA scan is taken, the region of interest (ROI) will be defined to analyze the LEM composition.
  Lumbar spine MRI will be also performed immediately after the 12-week intervention using a 1.5-T scanner (Achieva 1.5T; Philips Healthcare, Netherlands). The imaging protocol will include sagittal T2-weighted fast spin-echo imaging (repetition time, 3200 ms/echo; echo time, 100 ms; echo-train length, 20; section thickness, 4 mm; and field of view, 300 × 300 mm) and axial T2-weighted fast spin-echo imaging (repetition time, 3500 ms/echo; echo time, 100 ms; echo-train length, 20; section thickness, 4 mm; and field of view, 200 × 200 mm). Axial images (5 slices) will be obtained of each lumbar intervertebral level (T12/L1–L5/S1) parallel to the vertebral endplates. Three-dimensional segmentation of the LEM will be performed to measure the mean volume and signal intensity. The right and left LEM compartments will be separately segmented from the mid-disc level of T12/L1 to the mid-disc level of L4/5. A semi-automated random walk 3D segmentation algorithm, a magic cut tool for segmentation work of radiological software (AVIEW Research; Coreline, Seoul, South Korea) will be used to volumetrically segment the LEM.^[14]^ During the segmentation, an experienced radiologist will repeatedly modify the procedures and confirm the segmentation results. In this process, the ROI will be positioned at the muscle contour with care taken to avoid the accidental inclusion of subcutaneous fat or the muscle–fat interface. Bilateral LEM compartments will be combined to determine the mean signal intensity and volume. The mean signal intensity of the LEM reflects the intramuscular fat content because signal intensity increases as fat content increases.
3. *Conventional sarcopenic indices*
  A. Appendicular skeletal muscle mass (ASM): DEXA and bio-impedance analysis (InBody 720; Biospace, Seoul, South Korea) will be performed to analyze body composition, including lean body and fat masses. ASM will be calculated by summing the lean masses of the bilateral upper and lower extremities^[15]^ and standardized by being divided by the squared height value (ASM/Ht^2^ [kg/m^2^]).
  B. Handgrip strength: A handgrip dynamometer (T.K.K.5401; Takei Scientific Instruments, Tokyo, Japan)^[16]^ will be used to determine handgrip strength, as described previously.^[17]^ Briefly, while sitting in a straight-backed chair with their feet flat on the floor, the patients will be asked to adduct and neutrally rotate the shoulder, flex the elbow to 90°, and place the forearm in a neutral position with the wrist in 0–30° of extension and 0–15° of ulnar deviation. The subjects will be instructed to squeeze the handle as hard as possible for 3 seconds, and the maximum contraction force (kg) will be recorded.
  C. Performance: The Short Physical Performance Battery, which includes 3 objective physical function tests (ie, time taken to cover 4 m at a comfortable walking speed, time taken to stand from sitting in a chair 5 times without stopping, and ability to maintain balance for 10 seconds in 3 different foot positions at progressively more challenging levels), will be used to determine lower extremity function.^[18]^ A score of 0–4 will be used to grade the performance of each task, with higher scores indicating better lower extremity function.
4. *Spine specific outcomes*
  A. Spinal sagittal balance (SSB): For each participant, 1 lateral radiograph of the whole spine will be collected and digitized. All measurements will be performed using imaging software (INFINITT PACS M6; INFINITT Healthcare, Seoul, South Korea), as previously described.^[19,20]^ Briefly, the following spinopelvic radiographic parameters will be analyzed: sacral slope (SS), pelvic incidence (PI), pelvic tilt, lumbar lordosis (LL), TK, LL to PI ratio (LL/PI), PI-LL mismatch (difference between PI and LL), and sagittal vertical axis. PI-LL will be the primary SSB outcome.^[21]^
  B. Mobility-related activity: The Back Performance Scale (BPS), which consists of the sock test, pick-up test, roll-up test, fingertip-to-floor test, and lift test, will be used to assess trunk mobility. These 5 tests are associated with each other and have high internal consistency, implying that they share the ability to measure physical performance.^[22]^ The BPS sum score (0–15) is calculated by adding the individual scores of the 5 tests.
  C. Isometric endurance of the back: The Sorensen test, which is the most widely used test in published studies to evaluate the isometric endurance of the trunk extensor muscles, will be used to measure the duration of time a person can hold the unsupported upper body in a horizontal prone position with the lower body fixed to the examining table.^[23]^
5. *Other functional outcomes*
  A. Balance and fall risk: Each participant's balance and fall risk will be evaluated using the Berg Balance Scale (range: 0–56; lower scores indicate worse outcomes).^[24]^
  B. Quality of life: The Euro Quality of Life Questionnaire 5-dimensional classification (score range, 0–1; lower scores indicate worse outcomes) will be used to evaluate patients’ QoL.^[25]^
  C. Ability to perform activities of daily living (ADLs): The Korean version of the Modified Barthel index^[26]^ (score range, 0–100; lower scores indicate worse outcomes) and the Korean version of the Instrumental ADL (score range, 0–3; higher scores indicate worse outcomes) will be used to evaluate each subject's ability to perform ADLs.^[27]^
  D. Frailty: The Korean version of the FRAIL scale will be used to assess frailty using the fatigue, resistance, ambulation, illness, and loss of weight components (score range, 0–5; lower scores indicate worse outcomes).^[28]^

### 2.5 Data analysis

Data will be collected using a standardized data entry form and entered into the data management system. The intention-to-treat principle will be used for data analysis. Participant characteristics will be described using means and standard deviations for continuous data and frequencies and percentages for categorical data. To compare paired data between 2 different points, we will use repeated measures analysis of variance and Friedman tests for continuous and non-parametric data, respectively. Statistical significance will be defined as *P* values <.05. All statistical analyses will be performed using SPSS version 19.0 for Windows (IBM Corp., Chicago, IL, USA).

### 2.6 Sample size

We intended to perform the sample size calculation based on the difference in mean isometric back muscle strength or LEM mass. However, no data, let alone effect sizes, are available in the literature concerning isometric back muscle strength or LEM mass in general practices or hospitals. Therefore, we based our sample size calculation on feasibility and the number of participants in our previous cohort study.

### 2.7 Ethics and dissemination

This study protocol received approval from the institutional review board of Seoul Metropolitan Government Seoul National University (SMG-SNU) Boramae Medical Center (no. 10-2021-27). The study will be performed in accordance with the relevant guidelines of the Declaration of Helsinki, 1964, as amended in Tokyo, 1975; Venice, 1983; Hong Kong, 1989; and Somerset West, 1996.^[29]^ Written informed consent for all interventions and examinations will be obtained at patient admission. The ethics board will be informed of all serious adverse events and any unanticipated adverse effects that occur during the study. The study protocol has been registered at Clinicaltrials.gov and will be updated. Direct access to the source data will be provided for monitoring, audits, research ethics committee/institutional review board review, and regulatory authority inspections during and after the study. All patient information will be coded anonymously, with only the study team having access to the original data. The study results will be disseminated in peer-reviewed publications and conference presentations.

## 3 Discussion

Sarcopenia has become an important topic in geriatric medicine^[30]^ and has been registered in the disease code systems of countries worldwide since its recognition as a disease entity with the awarding of an International Classification of Diseases 10th edition, Clinical Modification (M62.84) code in September 2016.^[31]^ Almost all studies and clinical approaches to sarcopenia to date have been confined to the limb muscles. DEXA, a standard imaging test for measuring muscle mass, is unable to distinguish the skeletal muscles among the trunk muscles. Muscle strength and physical performance tests represented by handgrip strength and gait speed can easily evaluate the muscles of the upper and lower limbs.^[32]^ However, studies of the muscles around the spine, which are distributed around the trunk and significantly influence body function, remain very rare.

Both the atrophy of and fatty changes to the paraspinal muscles originate from sarcopenia and are known to be associated with functional disorders and chronic back pain. We have already termed this phenomenon “spinal sarcopenia” and established a small local cohort to collect relevant variables, including lumbar spine MRI, of community-dwelling elderly people.^[1]^ This prospective cohort study will continue for up to 4 more years to confirm the natural aging of the paraspinal muscles versus the limb muscles and to identify important variables of spinal sarcopenia and present related cutoff values.

Resistance exercises and protein supplementation are the first-line interventions for sarcopenia.^[7]^ Strong evidence supports the application of progressive resistance exercises suitable for older adults, as well as targeted protein intake and nutrition counseling.^[33,34]^ Therefore, it is necessary to confirm the clinical effects of spinal extension exercises and nutritional supplementation on spinal sarcopenia.

Through this pilot study, we intend to establish a resistance exercise and nutritional supplementation program that focuses on increasing LEM strength and confirm the feasibility and effectiveness of its 12-week delivery for treating spinal sarcopenia. This study is expected to increase interest in the concept of, and related research into, the development of interventions for treating spinal sarcopenia.

## Author contributions

**Conceptualization:** Sang Yoon Lee, Jae-Young Lim.

**Data curation:** Sang Yoon Lee, Jinhee Park, Dong Hyun Kim.

**Formal analysis:** Sang Yoon Lee, Dong Hyun Kim.

**Funding acquisition:** Sang Yoon Lee.

**Investigation:** Sang Yoon Lee, Jinhee Park, Dong Hyun Kim, Jae-Young Lim.

**Methodology:** Sang Yoon Lee, Jinhee Park, Dong Hyun Kim.

**Project administration:** Sang Yoon Lee, Jinhee Park, Dong Hyun Kim, Jae-Young Lim.

**Resources:** Jinhee Park.

**Software:** Dong Hyun Kim.

**Supervision:** Sang Yoon Lee, Jae-Young Lim.

**Validation:** Sang Yoon Lee.

**Visualization:** Sang Yoon Lee.

**Writing – original draft:** Sang Yoon Lee, Dong Hyun Kim.

**Writing – review & editing:** Sang Yoon Lee, Jinhee Park, Dong Hyun Kim, Jae-Young Lim.
